# Supplementary material for: Transcriptome and Metabolome Analysis of Selenium Treated Alfalfa Reveals Influence on Phenylpropanoid Biosynthesis to Enhance Growth
Source: Plants (Basel). 2023 May 19;12(10):2038. doi: 10.3390/plants12102038 (PMC10224443; doi:10.3390/plants12102038)
Supplement: Supplementary file 1 [file plants-12-02038-s001.zip › Figure S1-S5.pdf]

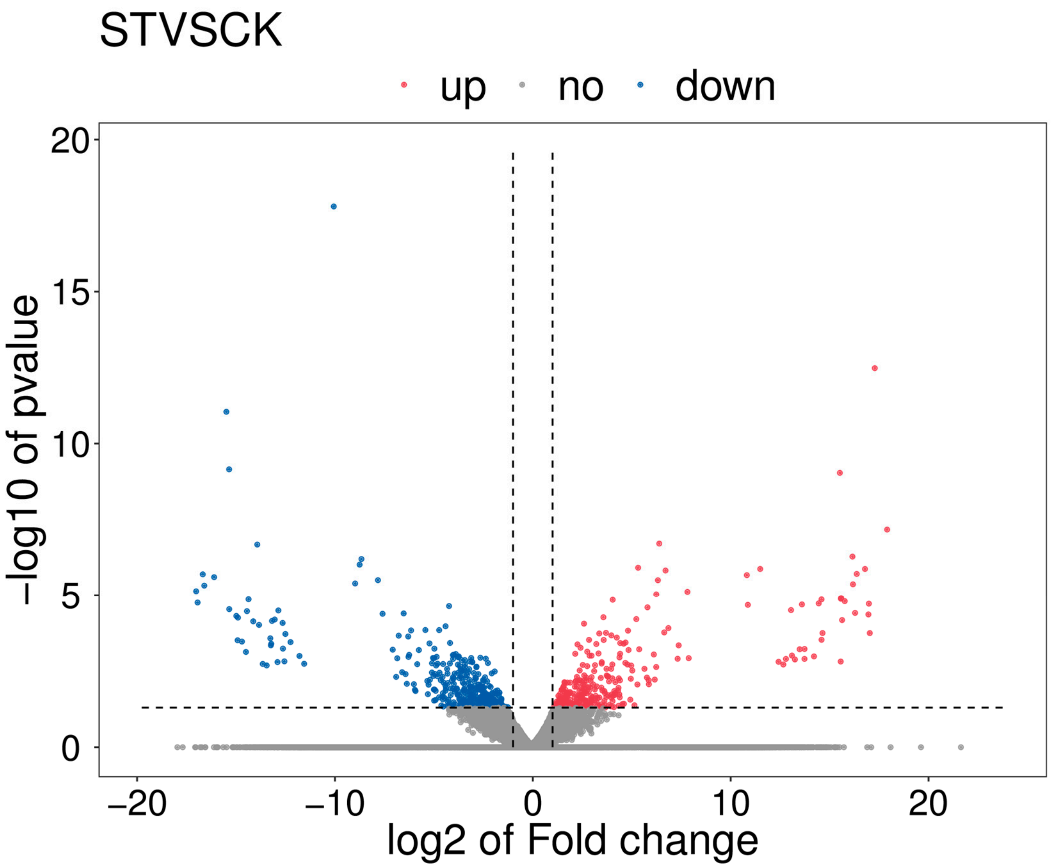

Figure S1: The volcano diagram of DEGs

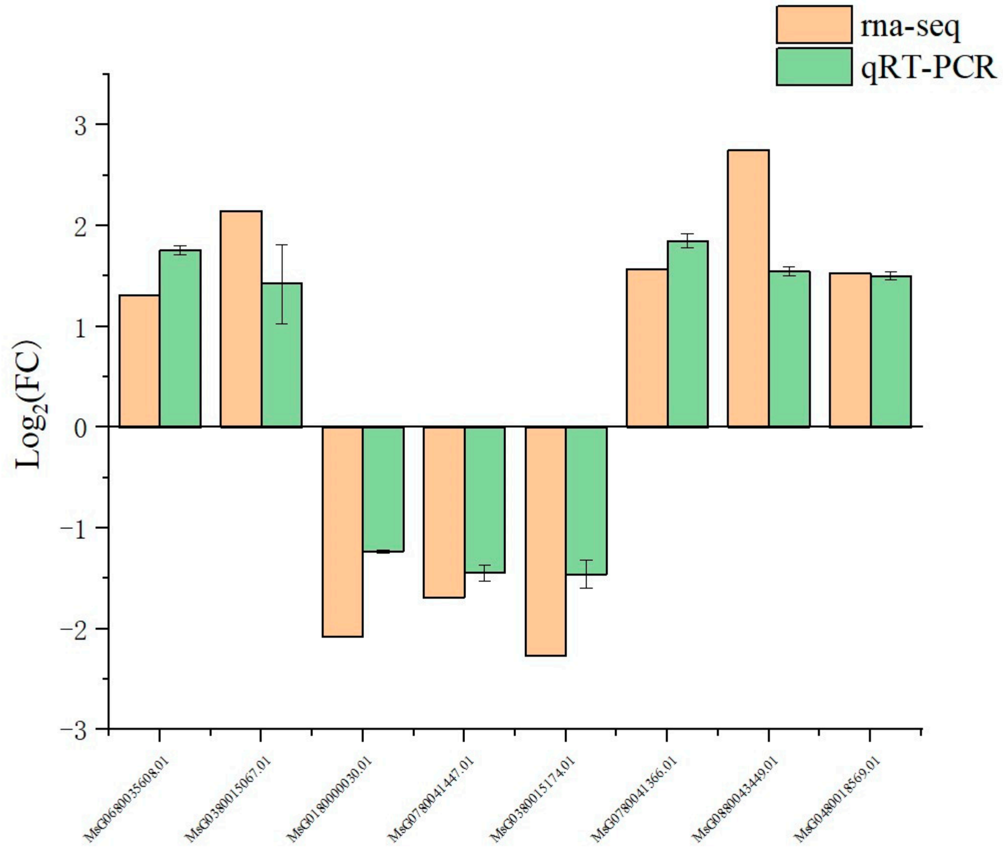

Figure S2: The qRT–PCR verification of selected genes

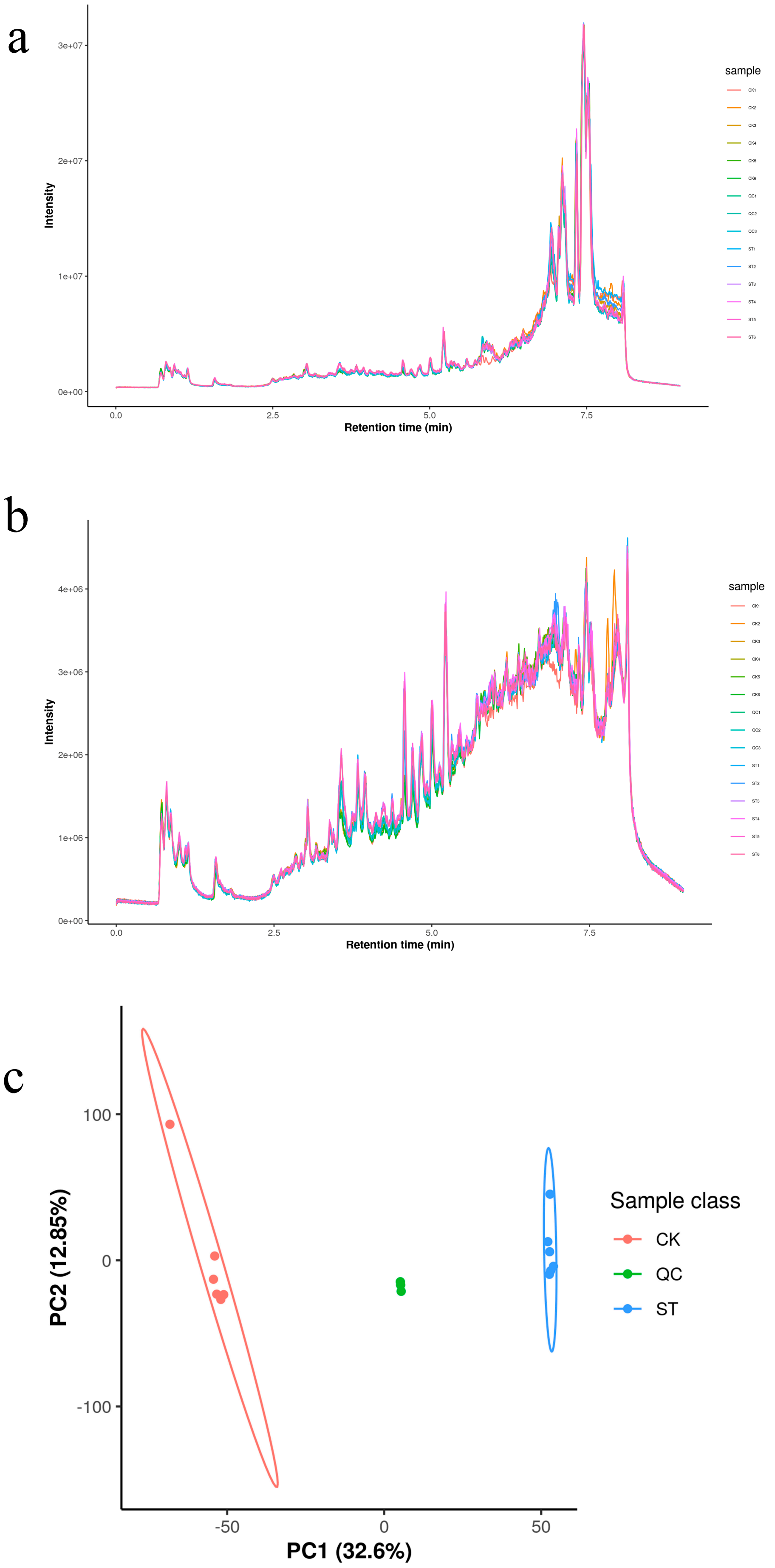
a
Intensity
Retention time (min)
sample
b
Intensity
Retention time (min)
sample
c
PC2 (12.85%)
PC1 (32.6%)
Sample class
CK
QC
ST

Figure S3: The positive (**a**) and negative (**b**) total ion flow chromatogram diagram and the PCA analysis (**c**) of alfalfa samples

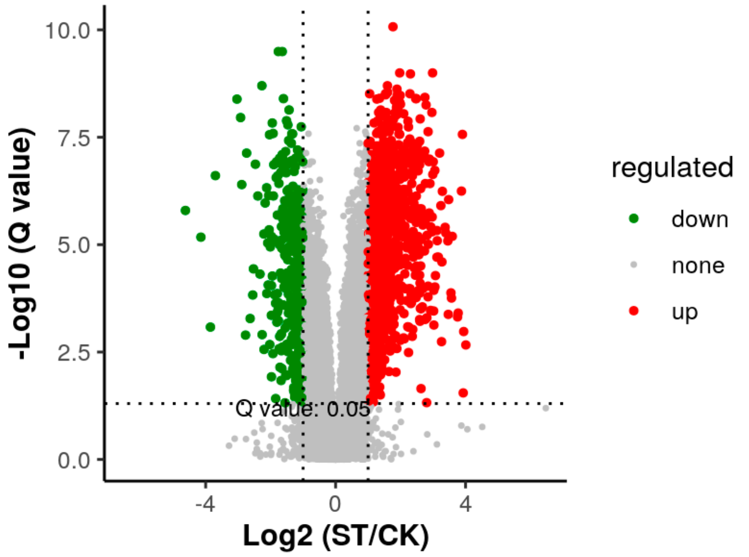

Figure S4: The volcano diagram of DEMs

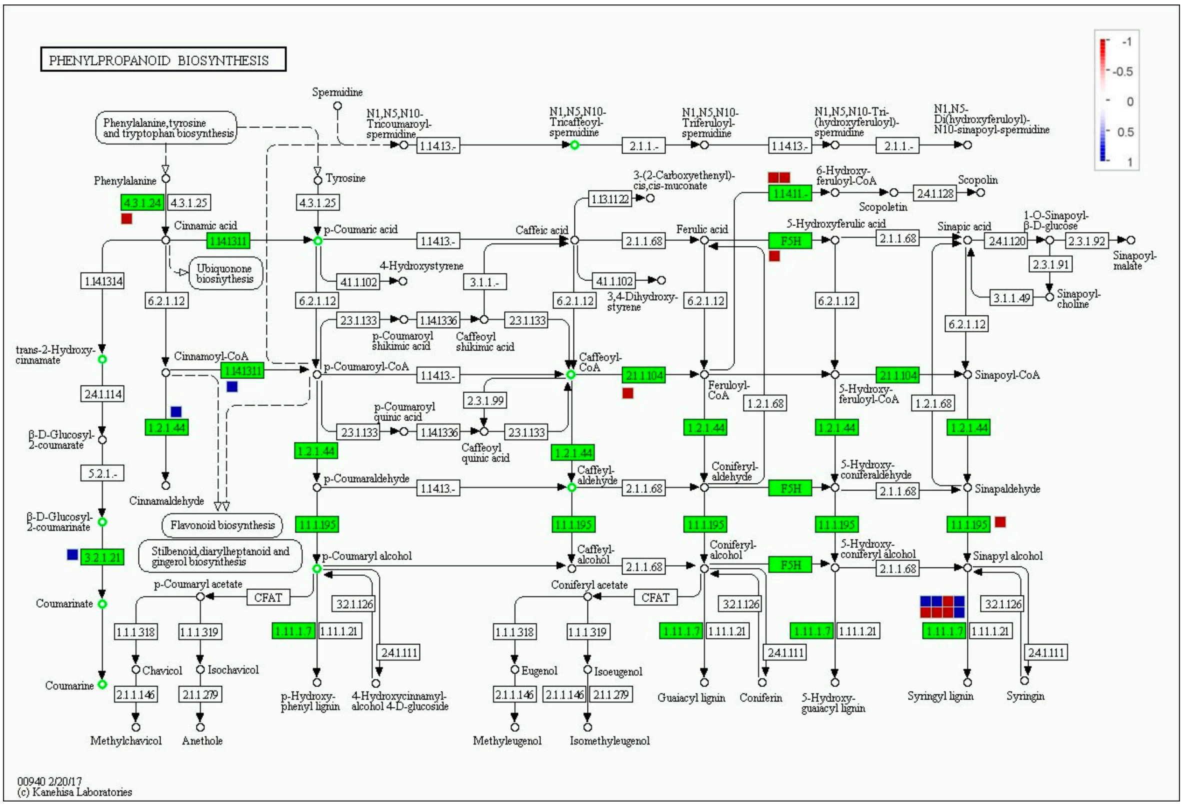

Figure S5: The DEGs and DEMs in phenylpropanoid biosynthesis pathways
