# Supplementary material for: Transcriptome and Metabolome Analysis of Selenium Treated Alfalfa Reveals Influence on Phenylpropanoid Biosynthesis to Enhance Growth
Source: Plants (Basel). 2023 May 19;12(10):2038. doi: 10.3390/plants12102038 (PMC10224443; doi:10.3390/plants12102038)
Supplement: Supplementary file 1 [file plants-12-02038-s001.zip › Table S1.pdf]

Table S1. Primers of 8 selected DEGs for qRT-PCR analysis

Gene id		Primer(5′-3′)
MsG0680035608.01	Forward	TGCTGGGTTTTCATGCGTATATTC
	Reverse	AACTTGAGGAGGCACTTGGATG
MsG0380015067.01	Forward	CTTCCCTATGCCGATGACCTA
	Reverse	AGCTAGTTCCTCAGTCCACCAAT
MsG0180000030.01	Forward	GACGCAGCAGCACCTCTACT
	Reverse	CCAAACGTTCCTTAGCCTCAG
MsG0780041447.01	Forward	TGCAAACCAGCTTCCAATGTAAC
	Reverse	GTCCACCTACAGCATAGTCAATACG
MsG0380015174.01	Forward	TGAATGCAAGAAAGGGAAGTGGAAG
	Reverse	CTACAAGATATCCGCAAATCACAGC
MsG0780041366.01.	Forward	GATGCTTCATTGCTCATCATCACC
	Reverse	GTTTCGGATTCTTTTGGTCGTTGA
MsG0880043449.01	Forward	GTGACATGGCTGGTGTGGTTATC
	Reverse	ATCCTTCTATTGCTGTTTGAACTGC
MsG0480018569.01	Forward	GGGCACACTCAGGGAGGTCAAC
	Reverse	TCATCGAGGATCTCAAGGCGTTC
GAPDH	Forward	GGCTGCCATCAAGGAGGAAT
	Reverse	TCCAAGCTCAGCCTCATCAAG
