# Supplementary material for: Transcriptome and Metabolome Analysis of Selenium Treated Alfalfa Reveals Influence on Phenylpropanoid Biosynthesis to Enhance Growth
Source: Plants (Basel). 2023 May 19;12(10):2038. doi: 10.3390/plants12102038 (PMC10224443; doi:10.3390/plants12102038)
Supplement: Supplementary file 1 [file plants-12-02038-s001.zip › Table S2.pdf]

**Table S2**. Integrative analysis of differential metabolites and DEGs

| ID of DEGs | Regulation | ID of DEMs | Regulation | KEGG |
|---|---|---|---|---|
| MsG0480020550.01 | up | pos-M653T209 | up | ko00520 |
| MsG0780036197.01 | up | pos-M148T52_1 | up | ko00430 |
| MsG0480021518.01 | up | pos-M147T286 | up | ko00940 |
| MsG0480021518.01 | up | pos-M165T285 | up | ko00940 |
| MsG0380016360.01 | up | pos-M291T263 | up | ko00380;ko00280;ko00310;ko00010;ko00410;ko00071;ko00620;ko00903;ko00330;ko00340;ko00053;ko00561 |
| MsG0380016360.01 | up | pos-M929T234 | up | ko00380;ko00280;ko00310;ko00010;ko00410;ko00071;ko00620;ko00903;ko00330;ko00340;ko00053;ko00561 |
| MsG0380016360.01 | up | pos-M170T178 | up | ko00380;ko00280;ko00310;ko00010;ko00410;ko00071;ko00620;ko00903;ko00330;ko00340;ko00053;ko00561 |
| MsG0380016360.01 | up | pos-M268T183 | up | ko00380;ko00280;ko00310;ko00010;ko00410;ko00071;ko00620;ko00903;ko00330;ko00340;ko00053;ko00561 |
| MsG0380016360.01 | up | pos-M170T178 | up | ko00380;ko00280;ko00310;ko00010;ko00410;ko00071;ko00620;ko00903;ko00330;ko00340;ko00053;ko00561 |
| MsG0380016360.01 | up | pos-M148T52_1 | up | ko00380;ko00280;ko00310;ko00010;ko00410;ko00071;ko00620;ko00903;ko00330;ko00340;ko00053;ko00561 |
| MsG0380016360.01 | up | pos-M272T184 | up | ko00380;ko00280;ko00310;ko00010;ko00410;ko00071;ko00620;ko00903;ko00330;ko00340;ko00053;ko00561 |
| MsG0380016360.01 | up | pos-M215T94 | up | ko00380;ko00280;ko00310;ko00010;ko00410;ko00071;ko00620;ko00903;ko00330;ko00340;ko00053;ko00561 |
| MsG0480019214.01 | up | pos-M148T52_1 | up | ko00040 |
| MsG0480019214.01 | up | pos-M215T94 | up | ko00040 |
| MsG0380016463.01 | up | pos-M139T177 | up | ko00071;ko00592;ko00010;ko00350 |
| MsG0380016463.01 | up | pos-M170T178 | up | ko00071;ko00592;ko00010;ko00350 |

| | | | | |
|---|---|---|---|---|
| MsG0780040733.01 | up | pos-M139T177 | up | ko04075;ko04016 |
| MsG0480021267.01 | up | pos-M653T209 | up | ko00500;ko00520 |
| MsG0380015362.01 | up | pos-M139T177 | up | ko04016;ko04075 |
| MsG0480021533.01 | up | pos-M170T178 | up | ko00220;ko00330;ko04626 |
| MsG0480021533.01 | up | pos-M148T52_1 | up | ko00220;ko00330;ko04626 |
| MsG0480021533.01 | up | pos-M272T184 | up | ko00220;ko00330;ko04626 |
| MsG0180003601.01 | up | pos-M147T286 | up | ko00940 |
| MsG0180003601.01 | up | pos-M165T285 | up | ko00940 |
| MsG0380012690.01 | up | pos-M233T320 | up | ko00620;ko00710 |
| MsG0880044676.01 | up | pos-M165T285 | up | ko00130 |
| MsG0880044676.01 | up | pos-M139T177 | up | ko00130 |
| MsG0480018671.01 | up | pos-M148T52_1 | up | ko00500;ko00460;ko00940 |
| MsG0480018671.01 | up | pos-M147T286 | up | ko00500;ko00460;ko00940 |
| MsG0480018671.01 | up | pos-M165T285 | up | ko00500;ko00460;ko00940 |
| MsG0280008343.01 | up | pos-M139T177 | up | ko04016;ko04075 |
| MsG0380013501.01 | up | pos-M170T178 | up | ko00750 |
| MsG0480018569.01 | up | pos-M139T177 | up | ko04075;ko04016 |
| MsG0480019215.01 | up | pos-M148T52_1 | up | ko00040 |
| MsG0480019215.01 | up | pos-M215T94 | up | ko00040 |
| MsG0480021524.01 | up | pos-M147T286 | up | ko00940 |
| MsG0480021524.01 | up | pos-M165T285 | up | ko00940 |
| MsG0780041613.01 | up | pos-M334T263 | up | ko04146;ko00590 |

| | | | | |
|---|---|---|---|---|
| MsG0280011263.01 | up | pos-M139T177 | up | ko04075 |
| MsG0480021433.01 | up | pos-M170T178 | up | ko00330;ko00380;ko00360 |
| MsG0480021433.01 | up | pos-M148T52_1 | up | ko00330;ko00380;ko00360 |
| MsG0480021433.01 | up | pos-M272T184 | up | ko00330;ko00380;ko00360 |
| MsG0480021433.01 | up | pos-M291T263 | up | ko00330;ko00380;ko00360 |
| MsG0480021433.01 | up | pos-M237T285 | up | ko00330;ko00380;ko00360 |
| MsG0480021433.01 | up | pos-M165T285 | up | ko00330;ko00380;ko00360 |
| MsG0480021433.01 | up | pos-M929T234 | up | ko00330;ko00380;ko00360 |
| MsG0380011489.01 | up | pos-M147T286 | up | ko00940 |
| MsG0380011489.01 | up | pos-M165T285 | up | ko00940 |
| MsG0080048387.01 | up | pos-M148T52_1 | up | ko00460 |
| MsG0880046345.01 | up | pos-M147T286 | up | ko00940 |
| MsG0880046345.01 | up | pos-M165T285 | up | ko00940 |
| MsG0180005984.01 | up | pos-M271T215 | up | ko00945;ko00941;ko00130;ko00360;ko00940 |
| MsG0180005984.01 | up | pos-M165T285 | up | ko00945;ko00941;ko00130;ko00360;ko00940 |
| MsG0180005984.01 | up | pos-M139T177 | up | ko00945;ko00941;ko00130;ko00360;ko00940 |
| MsG0180005984.01 | up | pos-M237T285 | up | ko00945;ko00941;ko00130;ko00360;ko00940 |
| MsG0180005984.01 | up | pos-M165T285 | up | ko00945;ko00941;ko00130;ko00360;ko00940 |
| MsG0180005984.01 | up | pos-M929T234 | up | ko00945;ko00941;ko00130;ko00360;ko00940 |
| MsG0180005984.01 | up | pos-M147T286 | up | ko00945;ko00941;ko00130;ko00360;ko00940 |
| MsG0180005984.01 | up | pos-M165T285 | up | ko00945;ko00941;ko00130;ko00360;ko00940 |
| MsG0780040107.01 | up | pos-M445T199 | down | ko00592 |

| | | | | |
|---|---|---|---|---|
| MsG0180 004680.01 | up | pos-M527T56 | down | ko00052 |
| MsG0480 021518.01 | up | pos-M649T272 | down | ko00940 |
| MsG0480 021518.01 | up | pos-M151T237 | down | ko00940 |
| MsG0480 021518.01 | up | pos-M349T201 | down | ko00940 |
| MsG0480 021518.01 | up | pos-M147T253 | down | ko00940 |
| MsG0480 018096.01 | up | pos-M233T374 | down | ko00909 |
| MsG0480 020076.01 | up | pos-M231T69 | down | ko00945 |
| MsG0380 016463.01 | up | pos-M445T199 | down | ko00071;ko00592;ko00010;ko00350 |
| MsG0680 031463.01 | up | pos-M284T449_3 | down | ko00906 |
| MsG0680 031463.01 | up | pos-M858T407 | down | ko00906 |
| MsG0580 024178.01 | up | pos-M445T199 | down | ko00592 |
| MsG0180 001827.01 | up | pos-M284T449_3 | down | ko00906 |
| MsG0180 001827.01 | up | pos-M858T407 | down | ko00906 |
| MsG0180 003601.01 | up | pos-M649T272 | down | ko00940 |
| MsG0180 003601.01 | up | pos-M151T237 | down | ko00940 |
| MsG0180 003601.01 | up | pos-M349T201 | down | ko00940 |
| MsG0180 003601.01 | up | pos-M147T253 | down | ko00940 |
| MsG0880 044676.01 | up | pos-M439T410 | down | ko00130 |
| MsG0380 012002.01 | up | pos-M415T191 | down | ko02010 |
| MsG0380 012002.01 | up | pos-M527T56 | down | ko02010 |
| MsG0380 016151.01 | up | pos-M307T300 | down | ko00061;ko04146;ko00071 |
| MsG0580 029922.01 | up | pos-M415T191 | down | ko02010 |

| | | | | |
|---|---|---|---|---|
| MsG0580029922.01 | up | pos-M527T56 | down | ko02010 |
| MsG0480018671.01 | up | pos-M649T272 | down | ko00500;ko00460;ko00940 |
| MsG0480018671.01 | up | pos-M151T237 | down | ko00500;ko00460;ko00940 |
| MsG0480018671.01 | up | pos-M349T201 | down | ko00500;ko00460;ko00940 |
| MsG0480018671.01 | up | pos-M147T253 | down | ko00500;ko00460;ko00940 |
| MsG0580028567.01 | up | pos-M231T69 | down | ko00945 |
| MsG0580024168.01 | up | pos-M445T199 | down | ko00592 |
| MsG0480021524.01 | up | pos-M649T272 | down | ko00940 |
| MsG0480021524.01 | up | pos-M151T237 | down | ko00940 |
| MsG0480021524.01 | up | pos-M349T201 | down | ko00940 |
| MsG0480021524.01 | up | pos-M147T253 | down | ko00940 |
| MsG0580024567.01 | up | pos-M284T449_3 | down | ko00906 |
| MsG0580024567.01 | up | pos-M858T407 | down | ko00906 |
| MsG0780041613.01 | up | pos-M359T420 | down | ko04146;ko00590 |
| MsG0780036262.01 | up | pos-M233T374 | down | ko00909 |
| MsG0480021433.01 | up | pos-M151T237 | down | ko00330;ko00380;ko00360 |
| MsG0380011489.01 | up | pos-M649T272 | down | ko00940 |
| MsG0380011489.01 | up | pos-M151T237 | down | ko00940 |
| MsG0380011489.01 | up | pos-M349T201 | down | ko00940 |
| MsG0380011489.01 | up | pos-M147T253 | down | ko00940 |
| MsG0880047256.01 | up | pos-M415T191 | down | ko02010 |
| MsG0880047256.01 | up | pos-M527T56 | down | ko02010 |

| | | | | |
|---|---|---|---|---|
| MsG0880046345.01 | up | pos-M649T272 | down | ko00940 |
| MsG0880046345.01 | up | pos-M151T237 | down | ko00940 |
| MsG0880046345.01 | up | pos-M349T201 | down | ko00940 |
| MsG0880046345.01 | up | pos-M147T253 | down | ko00940 |
| MsG0180005984.01 | up | pos-M231T69 | down | ko00945;ko00941;ko00130;ko00360;ko00940 |
| MsG0180005984.01 | up | pos-M433T253 | down | ko00945;ko00941;ko00130;ko00360;ko00940 |
| MsG0180005984.01 | up | pos-M439T410 | down | ko00945;ko00941;ko00130;ko00360;ko00940 |
| MsG0180005984.01 | up | pos-M151T237 | down | ko00945;ko00941;ko00130;ko00360;ko00940 |
| MsG0180005984.01 | up | pos-M649T272 | down | ko00945;ko00941;ko00130;ko00360;ko00940 |
| MsG0180005984.01 | up | pos-M151T237 | down | ko00945;ko00941;ko00130;ko00360;ko00940 |
| MsG0180005984.01 | up | pos-M349T201 | down | ko00945;ko00941;ko00130;ko00360;ko00940 |
| MsG0180005984.01 | up | pos-M147T253 | down | ko00945;ko00941;ko00130;ko00360;ko00940 |
| MsG0780039574.01 | down | pos-M433T253 | down | ko00943 |
| MsG0580030181.01 | down | pos-M649T272 | down | ko00940;ko00360 |
| MsG0580030181.01 | down | pos-M151T237 | down | ko00940;ko00360 |
| MsG0580030181.01 | down | pos-M349T201 | down | ko00940;ko00360 |
| MsG0580030181.01 | down | pos-M147T253 | down | ko00940;ko00360 |
| MsG0580030181.01 | down | pos-M151T237 | down | ko00940;ko00360 |
| MsG0880045881.01 | down | pos-M433T253 | down | ko00941;ko00945;ko00360;ko00940 |
| MsG0880045881.01 | down | pos-M231T69 | down | ko00941;ko00945;ko00360;ko00940 |
| MsG0880045881.01 | down | pos-M151T237 | down | ko00941;ko00945;ko00360;ko00940 |
| MsG0880045881.01 | down | pos-M649T272 | down | ko00941;ko00945;ko00360;ko00940 |

| | | | | |
|---|---|---|---|---|
| MsG0880045881.01 | down | pos-M151T237 | down | ko00941;ko00945;ko00360;ko00940 |
| MsG0880045881.01 | down | pos-M349T201 | down | ko00941;ko00945;ko00360;ko00940 |
| MsG0880045881.01 | down | pos-M147T253 | down | ko00941;ko00945;ko00360;ko00940 |
| MsG0180000030.01 | down | pos-M649T272 | down | ko00940 |
| MsG0180000030.01 | down | pos-M151T237 | down | ko00940 |
| MsG0180000030.01 | down | pos-M349T201 | down | ko00940 |
| MsG0180000030.01 | down | pos-M147T253 | down | ko00940 |
| MsG0180005051.01 | down | pos-M415T191 | down | ko02010 |
| MsG0180005051.01 | down | pos-M527T56 | down | ko02010 |
| MsG0280008072.01 | down | pos-M433T253 | down | ko00943 |
| MsG0780038491.01 | down | pos-M649T272 | down | ko00940 |
| MsG0780038491.01 | down | pos-M151T237 | down | ko00940 |
| MsG0780038491.01 | down | pos-M349T201 | down | ko00940 |
| MsG0780038491.01 | down | pos-M147T253 | down | ko00940 |
| MsG0580028020.01 | down | pos-M439T410 | down | ko00130 |
| MsG0380013348.01 | down | pos-M649T272 | down | ko00940 |
| MsG0380013348.01 | down | pos-M151T237 | down | ko00940 |
| MsG0380013348.01 | down | pos-M349T201 | down | ko00940 |
| MsG0380013348.01 | down | pos-M147T253 | down | ko00940 |
| MsG0380016745.01 | down | pos-M649T272 | down | ko00940 |
| MsG0380016745.01 | down | pos-M151T237 | down | ko00940 |
| MsG0380016745.01 | down | pos-M349T201 | down | ko00940 |

| | | | | |
|---|---|---|---|---|
| MsG0380016745.01 | down | pos-M147T253 | down | ko00940 |
| MsG0380014934.01 | down | pos-M649T272 | down | ko00940 |
| MsG0380014934.01 | down | pos-M151T237 | down | ko00940 |
| MsG0380014934.01 | down | pos-M349T201 | down | ko00940 |
| MsG0380014934.01 | down | pos-M147T253 | down | ko00940 |
| MsG0480018433.01 | down | pos-M415T191 | down | ko02010 |
| MsG0480018433.01 | down | pos-M527T56 | down | ko02010 |
| MsG0480020786.01 | down | pos-M307T300 | down | ko00333;ko01040;ko00061;ko00780 |
| MsG0480020786.01 | down | pos-M307T300 | down | ko00333;ko01040;ko00061;ko00780 |
| MsG0680030961.01 | down | pos-M433T253 | down | ko00943;ko00944 |
| MsG0680030961.01 | down | pos-M579T241 | down | ko00943;ko00944 |
| MsG0680030961.01 | down | pos-M433T253 | down | ko00943;ko00944 |
| MsG0580030055.01 | down | pos-M527T56 | down | ko00052 |
| MsG0480022309.01 | down | pos-M433T253 | down | ko00943 |
| MsG0580025234.01 | down | pos-M649T272 | down | ko00940 |
| MsG0580025234.01 | down | pos-M151T237 | down | ko00940 |
| MsG0580025234.01 | down | pos-M349T201 | down | ko00940 |
| MsG0580025234.01 | down | pos-M147T253 | down | ko00940 |
| MsG0380017247.01 | down | pos-M345T206 | down | ko00770;ko00290;ko00270;ko00280 |
| MsG0380017247.01 | down | pos-M340T206 | down | ko00770;ko00290;ko00270;ko00280 |
| MsG0380017247.01 | down | pos-M181T206 | down | ko00770;ko00290;ko00270;ko00280 |
| MsG0480022835.01 | down | pos-M433T253 | down | ko00943 |

| | | | | |
|---|---|---|---|---|
| MsG0580030022.01 | down | pos-M527T56 | down | ko00052 |
| MsG0380012312.01 | down | pos-M415T191 | down | ko02010 |
| MsG0380012312.01 | down | pos-M527T56 | down | ko02010 |
| MsG0880042881.01 | down | pos-M433T253 | down | ko00941 |
| MsG0680032461.01 | down | pos-M649T272 | down | ko00940 |
| MsG0680032461.01 | down | pos-M151T237 | down | ko00940 |
| MsG0680032461.01 | down | pos-M349T201 | down | ko00940 |
| MsG0680032461.01 | down | pos-M147T253 | down | ko00940 |
| MsG0180000436.01 | down | pos-M527T56 | down | ko00052;ko00500 |
| MsG0280007686.01 | down | pos-M649T272 | down | ko00940 |
| MsG0280007686.01 | down | pos-M151T237 | down | ko00940 |
| MsG0280007686.01 | down | pos-M349T201 | down | ko00940 |
| MsG0280007686.01 | down | pos-M147T253 | down | ko00940 |
| MsG0580025965.01 | down | pos-M433T253 | down | ko00941 |
| MsG0580025922.01 | down | pos-M345T206 | down | ko00770;ko00290;ko00280;ko00270 |
| MsG0580025922.01 | down | pos-M340T206 | down | ko00770;ko00290;ko00280;ko00270 |
| MsG0580025922.01 | down | pos-M181T206 | down | ko00770;ko00290;ko00280;ko00270 |
| MsG0480018440.01 | down | pos-M291T281 | down | ko00904 |
| MsG0480018440.01 | down | pos-M307T300 | down | ko00904 |
| MsG0180000437.01 | down | pos-M527T56 | down | ko00052;ko00500 |
| MsG0780036575.01 | down | pos-M433T253 | down | ko00943 |
| MsG0780039574.01 | down | pos-M301T296 | up | ko00943 |

| | | | | |
|---|---|---|---|---|
| MsG0780039574.01 | down | pos-M271T215 | up | ko00943 |
| MsG0780039574.01 | down | pos-M286T448_6 | up | ko00943 |
| MsG0580030181.01 | down | pos-M147T286 | up | ko00940;ko00360 |
| MsG0580030181.01 | down | pos-M165T285 | up | ko00940;ko00360 |
| MsG0580030181.01 | down | pos-M237T285 | up | ko00940;ko00360 |
| MsG0580030181.01 | down | pos-M165T285 | up | ko00940;ko00360 |
| MsG0580030181.01 | down | pos-M929T234 | up | ko00940;ko00360 |
| MsG0280006550.01 | down | pos-M139T177 | up | ko04075;ko04016;ko04626 |
| MsG0880045881.01 | down | pos-M271T215 | up | ko00941;ko00945;ko00360;ko00940 |
| MsG0880045881.01 | down | pos-M237T285 | up | ko00941;ko00945;ko00360;ko00940 |
| MsG0880045881.01 | down | pos-M165T285 | up | ko00941;ko00945;ko00360;ko00940 |
| MsG0880045881.01 | down | pos-M929T234 | up | ko00941;ko00945;ko00360;ko00940 |
| MsG0880045881.01 | down | pos-M147T286 | up | ko00941;ko00945;ko00360;ko00940 |
| MsG0880045881.01 | down | pos-M165T285 | up | ko00941;ko00945;ko00360;ko00940 |
| MsG0180000030.01 | down | pos-M147T286 | up | ko00940 |
| MsG0180000030.01 | down | pos-M165T285 | up | ko00940 |
| MsG0380018070.01 | down | pos-M653T209 | up | ko00520;ko04016 |
| MsG0780041428.01 | down | pos-M139T177 | up | ko04075;ko04712 |
| MsG0280008072.01 | down | pos-M301T296 | up | ko00943 |
| MsG0280008072.01 | down | pos-M271T215 | up | ko00943 |
| MsG0280008072.01 | down | pos-M286T448_6 | up | ko00943 |
| MsG0880042495.01 | down | pos-M215T94 | up | ko00562;ko00053 |

| | | | | |
|---|---|---|---|---|
| MsG0780036260.01 | down | pos-M653T209 | up | ko00520;ko04016 |
| MsG0680030315.01 | down | pos-M215T94 | up | ko00053;ko00520;ko00040 |
| MsG0680030315.01 | down | pos-M653T209 | up | ko00053;ko00520;ko00040 |
| MsG0680030315.01 | down | pos-M148T52_1 | up | ko00053;ko00520;ko00040 |
| MsG0680030315.01 | down | pos-M215T94 | up | ko00053;ko00520;ko00040 |
| MsG0780038491.01 | down | pos-M147T286 | up | ko00940 |
| MsG0780038491.01 | down | pos-M165T285 | up | ko00940 |
| MsG0580028020.01 | down | pos-M165T285 | up | ko00130 |
| MsG0580028020.01 | down | pos-M139T177 | up | ko00130 |
| MsG0180000751.01 | down | pos-M653T209 | up | ko00520 |
| MsG0380013348.01 | down | pos-M147T286 | up | ko00940 |
| MsG0380013348.01 | down | pos-M165T285 | up | ko00940 |
| MsG0880046970.01 | down | pos-M233T320 | up | ko00710;ko00620 |
| MsG0380016745.01 | down | pos-M147T286 | up | ko00940 |
| MsG0380016745.01 | down | pos-M165T285 | up | ko00940 |
| MsG0680031118.01 | down | pos-M139T177 | up | ko00260;ko00400 |
| MsG0380014934.01 | down | pos-M147T286 | up | ko00940 |
| MsG0380014934.01 | down | pos-M165T285 | up | ko00940 |
| MsG0080048889.01 | down | pos-M148T52_1 | up | ko00040 |
| MsG0080048889.01 | down | pos-M215T94 | up | ko00040 |
| MsG0880045572.01 | down | pos-M653T209 | up | ko00520;ko04016 |
| MsG0880047320.01 | down | pos-M291T263 | up | ko00380 |

| | | | | |
|---|---|---|---|---|
| MsG0680031451.01 | down | pos-M653T209 | up | ko00520 |
| MsG0680030961.01 | down | pos-M301T296 | up | ko00943;ko00944 |
| MsG0680030961.01 | down | pos-M271T215 | up | ko00943;ko00944 |
| MsG0680030961.01 | down | pos-M286T448_6 | up | ko00943;ko00944 |
| MsG0680030961.01 | down | pos-M301T296 | up | ko00943;ko00944 |
| MsG0680030961.01 | down | pos-M635T214_2 | up | ko00943;ko00944 |
| MsG0480022309.01 | down | pos-M301T296 | up | ko00943 |
| MsG0480022309.01 | down | pos-M271T215 | up | ko00943 |
| MsG0480022309.01 | down | pos-M286T448_6 | up | ko00943 |
| MsG0580025234.01 | down | pos-M147T286 | up | ko00940 |
| MsG0580025234.01 | down | pos-M165T285 | up | ko00940 |
| MsG0380017247.01 | down | pos-M244T263 | up | ko00770;ko00290;ko00270;ko00280 |
| MsG0380017247.01 | down | pos-M148T52_1 | up | ko00770;ko00290;ko00270;ko00280 |
| MsG0380017247.01 | down | pos-M929T234 | up | ko00770;ko00290;ko00270;ko00280 |
| MsG0480022835.01 | down | pos-M301T296 | up | ko00943 |
| MsG0480022835.01 | down | pos-M271T215 | up | ko00943 |
| MsG0480022835.01 | down | pos-M286T448_6 | up | ko00943 |
| MsG0880045571.01 | down | pos-M653T209 | up | ko04016;ko00520 |
| MsG0880042881.01 | down | pos-M271T215 | up | ko00941 |
| MsG0280006552.01 | down | pos-M139T177 | up | ko04016;ko04626;ko04075 |
| MsG0680032461.01 | down | pos-M147T286 | up | ko00940 |
| MsG0680032461.01 | down | pos-M165T285 | up | ko00940 |

| | | | | |
|---|---|---|---|---|
| MsG0280007686.01 | down | pos-M147T286 | up | ko00940 |
| MsG0280007686.01 | down | pos-M165T285 | up | ko00940 |
| MsG0580026720.01 | down | pos-M653T209 | up | ko00520;ko00051;ko00500 |
| MsG0580025965.01 | down | pos-M271T215 | up | ko00941 |
| MsG0580025922.01 | down | pos-M244T263 | up | ko00770;ko00290;ko00280;ko00270 |
| MsG0580025922.01 | down | pos-M148T52_1 | up | ko00770;ko00290;ko00280;ko00270 |
| MsG0580025922.01 | down | pos-M929T234 | up | ko00770;ko00290;ko00280;ko00270 |
| MsG0480018440.01 | down | pos-M334T263 | up | ko00904 |
| MsG0780038305.01 | down | pos-M148T52_1 | up | ko00040 |
| MsG0780038305.01 | down | pos-M215T94 | up | ko00040 |
| MsG0280006553.01 | down | pos-M139T177 | up | ko04626;ko04016;ko04075 |
| MsG0780036575.01 | down | pos-M301T296 | up | ko00943 |
| MsG0780036575.01 | down | pos-M271T215 | up | ko00943 |
| MsG0780036575.01 | down | pos-M286T448_6 | up | ko00943 |
| MsG0180002935.01 | down | pos-M139T177 | up | ko04075 |
