# Supplementary material for: Transcriptome and Metabolome Analysis of Selenium Treated Alfalfa Reveals Influence on Phenylpropanoid Biosynthesis to Enhance Growth
Source: Plants (Basel). 2023 May 19;12(10):2038. doi: 10.3390/plants12102038 (PMC10224443; doi:10.3390/plants12102038)
Supplement: Supplementary file 1 [file plants-12-02038-s001.zip › Table S3.pdf]

Table S3. Genes in the phenylpropanoid biosynthesis pathway

Gene id	EC	Description	Regulation	Log2(FC)
MsG0480021518.01	EC:1.11.1.7	putative peroxidase	up	14.59
MsG0480021524.01	EC:1.11.1.7	putative peroxidase	up	13.25
MsG0180003601.01	EC:1.11.1.7	polyubiquitin peroxidase 21	up	2.75
MsG0880046345.01	EC:1.11.1.7	Gag-Pol polyprotein	up	3.72
MsG0380011489.01	EC:1.2.1.44	cinnamoyl-CoA reductase 1	up	1.45
MsG0480018671.01	EC:3.2.1.21	beta-glucosidase 11 isoform X1	up	2.35
MsG0180005984.01	EC:1.14.13.11	cytochrome P450 CYP73A100	up	2.96
MsG0680032461.01	EC:1.11.1.7	putative peroxidase	down	-6.78
MsG0380013348.01	EC:1.14.11	putative oxoglutarate	down	-3.3
MsG0180000030.01	EC:1.1.1.195	probable cinnamyl alcohol dehydrogenase 6	down	-2.08
MsG0380016745.01	EC:1.11.1.7	peroxidase P7	down	-3.79
MsG0280007686.01	EC:1.11.1.7	peroxidase	down	-2.05
MsG0580025234.01	EC:1.14	ferulate 5-hydroxylase	down	-3.05
MsG0380014934.01	EC:1.11.1.7	cationic peroxidase 1	down	-3.19
MsG0780038491.01	EC:1.14.11	1-aminocyclopropane-1-carboxylate oxidase homolog 1 isoform X1	down	-2.77
MsG0580030181.01	EC:4.3.1.24	phenylalanine ammonia-lyase class 3	down	-2.01
MsG0880045881.01	EC:2.1.1.104	caffeoyl-CoA O-methyltransferase 5	down	-4.56
