# Supplementary material for: Transcriptome and Metabolome Analysis of Selenium Treated Alfalfa Reveals Influence on Phenylpropanoid Biosynthesis to Enhance Growth
Source: Plants (Basel). 2023 May 19;12(10):2038. doi: 10.3390/plants12102038 (PMC10224443; doi:10.3390/plants12102038)
Supplement: Supplementary file 1 [file plants-12-02038-s001.zip › Table S4.pdf]

Table S4. Metabolite in phenylpropanoid biosynthesis pathway

Feature id	Metabolite	Regulation	VIP
pos-M929T234	(S)-3-Hydroxy-3-methylglutaryl-CoA	up	2.37
pos-M165T285	Phenylpyruvate	up	2.12
pos-M147T286	Coumarin A	up	2.14
pos-M271T215	Apigenin	up	2.05
pos-M237T285	Cymoxanil	up	2.26
pos-M148T52_1	L-Glutamate	up	1.93
pos-M139T177	4-Hydroxybenzoate	up	1.78
pos-M231T69	Piceatannol	down	2.21
pos-M649T272	N1,N5,N10-Tricaffeoyl spermidine	down	2.4
pos-M147T253	Coumarin B	down	2.44
pos-M433T253	Isovitexin	down	2.63
pos-M151T237	Tolylacetate	down	1.79
pos-M349T201	p-Coumaroyl-D-glucose	down	1.96
pos-M439T410	γ-Tocopherol	down	1.65
