# Supplementary material for: Transcriptome and Metabolome Analysis of Selenium Treated Alfalfa Reveals Influence on Phenylpropanoid Biosynthesis to Enhance Growth
Source: Plants (Basel). 2023 May 19;12(10):2038. doi: 10.3390/plants12102038 (PMC10224443; doi:10.3390/plants12102038)
Supplement: Supplementary file 1 [file plants-12-02038-s001.zip › Table S5.pdf]

Table S5. Identification of basic physicochemical properties of genes in phenylpropanoid biosynthesis pathway

Gene id	Chromosome: location/bp	Number of amino acids	Molecular weight/u	Isoelectric point	Instability index	Aliphatic index	Grand average of hydropathicity
MsG0880045881.01	8:65938326-65940246	246	27715.98	5.5	40.37	101.91	-0.123
MsG0180000030.01	1:508395-511538	278	30529.17	5.8	36.4	95.72	-0.017
MsG0480021518.01	4:58992075-58993350	320	34379.02	8.34	32.22	85.41	-0.011
MsG0780038491.01	7:47642511-47645114	373	42130.05	5.7	35.98	93.75	-0.233
MsG0380013348.01	3:35640148-35640669	173	19664.47	5.01	35.68	103.06	-0.094
MsG0380016745.01	3:87338418-87343610	97	10228.32	7.82	11.77	66.39	-0.642
MsG0380014934.01	3:62730558-62732167	256	26843.84	5.34	23.66	73.55	-0.17
MsG0180003601.01	1:65026421-65033431	100	11449.28	8.75	41	104.3	0.247
MsG0580025234.01	5:15672068-15675816	519	58649.14	6.01	40.13	93.04	-0.105
MsG0680032461.01	6:41833043-41838048	352	38505.79	8.36	32.58	83.64	-0.16
MsG0280007686.01	2:19198463-19201765	319	35309.67	9.07	33.65	93.82	-0.083
MsG0480021524.01	4:59036368-59047267	364	40658.68	8.1	30.87	73.46	-0.484

MsG038001148 9.01	3:201178-209562	308	33884.88	8.12	30.35	94.03	-0.069
MsG088004634 5.01	8:72258351-72259115	254	28248.3	7.08	44.68	73.7	-0.228
MsG058003018 1.01	5:107912337-107914508	536	58955.57	5.91	37.3	96.62	-0.081
MsG048001867 1.01	4:8451791-8458805	306	34645.55	5.91	34.57	80.88	-0.195
MsG018000598 4.01	1:98481320-98486933	417	47492.88	7.06	39.34	92.28	-0.224
